# The first trimester human placenta responds to Zika virus infection inducing an interferon (IFN) and antiviral interferon stimulated gene (ISG) response

**DOI:** 10.1186/s12985-025-02729-3

**Published:** 2025-04-19

**Authors:** Kylie H. Van der Hoek, Tanja Jankovic-Karasoulos, Dylan McCullough, Rosa C. Coldbeck-Shackley, Nicholas S. Eyre, Claire T. Roberts, Michael R. Beard

**Affiliations:** 1 Research Centre for Infectious Diseases, The University of Adelaide, Adelaide, SA 5005 Australia; 2 School of Biological Sciences, The University of Adelaide, Adelaide, SA 5005 Australia; 3 University of Adelaide, The Robinson Research Institute, Adelaide, SA 5005 Australia; 4 Flinders University, College of Medicine and Public Health, Flinders Health and Medical Research Institute, Adelaide, SA 5005 Australia; 5 Department of Molecular and Biomedical Science and Research Centre for Infectious Diseases, University of Adelaide, Adelaide, SA 5005 Australia

**Keywords:** Zika virus, Placenta, Trophoblast, First trimester pregnancy, Interferon, Interferon lambda, Interferon stimulated genes

## Abstract

**Background:**

Zika virus (ZIKV) is a positive-strand RNA virus of the Flaviviridae family. Maternal ZIKV infection during pregnancy can spread to the placenta and fetus causing severe neurological defects and infants born with microcephaly. Here, we investigated ZIKV infection and the cellular innate antiviral immune response in first trimester human placental explant cultures and isolated primary villus cytotrophoblasts (CTBs).

**Methods:**

Placentas were obtained with informed consent from women undergoing elective pregnancy termination and either cultured as placental explants or used to isolate primary CTBs. Explants and CTBs were both infected with ZIKV (PRVABC59), and samples evaluated for infection by qRT-PCR, viral plaque and ELISA assays, and immunohistochemical or immunocytochemical staining.

**Results:**

We demonstrate robust infection and production of ZIKV in placental explant and CTB cultures. Both displayed delayed upregulation of interferons (IFN), most notably IFNβ and IFNλ2/3, and a panel of interferon stimulated genes (ISG) *(IFI6*,* IFIT1*,* IFIT2*,* IFITM1*,* ISG15*,* MX1*,* RSAD*). Stimulation of explants and CTBs with the dsRNA mimic poly(I: C), caused immediate *IFN* and *ISG* upregulation, demonstrating the first trimester placenta is innate immune competent. This suggests that either ZIKV blocks the early innate response, or the placental response is inherently hindered.

**Conclusion:**

Together these data show that first trimester placenta is susceptible to ZIKV infection which induces a delayed type III IFN antiviral response. This delay likely creates an environment favourable to ZIKV replication and dissemination across the early gestation placenta to fetal tissue, causing pathologies associated with congenital ZIKV syndrome.

## Introduction

*Zika virus* (ZIKV) is a mosquito-borne *orthoflavivirus* which, unlike other Flaviviridae family members, can be transmitted sexually and vertically to infect the fetus. Most ZIKV infections are asymptomatic or associated with mild symptoms (fever, rash and conjunctivitis). However, during the 2016 Brazilian outbreak ZIKV infection during pregnancy was associated with detrimental impacts on fetal development. It is now established that infection during pregnancy, particularly in the first trimester, can result in infection of the placenta and fetus, causing spontaneous abortion, severe neurological defects and microcephaly [1–3]. Furthermore, babies delivered by mothers infected during pregnancy, with no obvious pathologies at birth, on follow-up can display significant neurodevelopmental anomalies [4–6].

In response to viral infection, the host mounts an innate immune response to control and eliminate the virus. This is initiated by recognition of virus through host cytoplasmic (RIG-I, MDA5) and membrane bound pattern recognition receptors (TLRs) that initiate the production of interferons (IFN) and subsequent expression of hundreds of interferon stimulated genes (ISGs), that inhibit viral replication and drive host inflammatory processes to prevent viral spread. The importance of this response is corroborated by evidence that most viruses, including ZIKV, have evolved mechanisms to evade or inactivate this innate response [7], including in placental cell lines [8, 9]. However, these cell lines may not accurately reflect in vivo first trimester placental innate responses. The human placenta is a transient organ of embryonic origin, responsible for nutrient and gas exchange between the mother and the fetus and orchestrating maternal adaptations to pregnancy. The outermost barrier of the placenta, the multinucleated syncytiotrophoblast (STB), is derived from proliferation and fusion of the underlying cytotrophoblast (CTB) cells. In the first trimester, placental villus CTBs also differentiate into extravillous cytotrophoblast (EVT) that invade the maternal endometrium anchoring the developing placenta to maternal decidual uterine tissue. The cellular antiviral response of the first trimester placenta to infection is not well characterised due to difficulties in obtaining appropriate tissue samples. Previous research has however demonstrated, infection of both first trimester (6–12 weeks) and second trimester (19–21 weeks) placental tissues with ZIKV, and shown reduced levels of IFN production and increased ZIKV replication early in gestation when compared to mid-gestation [10]. Moreover, comparison of primary trophoblasts from term placenta with trophoblasts generated in vitro from embryonic stem cells reveal the latter were more susceptible to ZIKV infection, lacked expression of IFN receptor subunits, and had a much lower basal expression of ISGs required to restrict virus infection [11]. Since, our understanding of the capacity of the first trimester placenta to respond to viral infection is lacking it was our aim to examine the innate immune response to ZIKV infection in first trimester human placental explants and isolated trophoblasts.

## Methods

### Human ethics approval for placental collection

First trimester (7–12 weeks gestation) placentas were obtained with written informed consent from women undergoing elective pregnancy terminations at the Pregnancy Advisory Centre (PAC), Woodville, South Australia. Ethics approval was granted by the Queen Elizabeth Hospital Human Research Ethics Committee (HREC/16/TQEH/33). Pregnancies from which placenta samples were donated were all deemed to be uncomplicated as far as could be ascertained. All terminations were elective for non-medical reasons with a dating ultrasound prior to the procedure that confirmed a fetal heartbeat. All Placentas were obtained within minutes of termination, non-villous tissue was removed and remaining villous tissue washed and transported to the laboratory in PBS on ice.

### Cell and placental explant culture conditions

All mammalian cells and human placental explant tissues were cultured at 37 °C (5%CO_2_/21% O_2_) with 10%FCS (Corning Life Sciences), Penicillin (100U/ml) and Streptomycin (100 mg/ml) (Invitrogen) added to the medium specified for each cell type. Vero and Huh7.5 cells were cultured in Hepes buffered DMEM (Gibco). C636 insect cells were cultured at 28 °C in Basal Medium Eagle (Gibco) with addition of 1xMEM non-essential amino acid solution, 1mM sodium pyruvate (Gibco) and 1% glutamax supplement (Gibco). Placental villus explants and CTBs were cultured in DMEM high glucose (Invitrogen 12430) with addition of Fungizone (Amphotericin,0.25 µg) (ThermoFisher), while in CTB cultures 1%Glutamax Supplement (Gibco) was also added.

### Virus propagation and quantification

*Zika* virus (ZIKV)(PRVABC59-Puerto Rico, 2015, #KU501215) and *Dengue* virus (DENV) (pFK-DV, DENV-2 strain 16681 [12], were propagated and quantified as described previously [8, 12–15]. Briefly, Vero cells were infected with serially diluted virus containing supernatants and overlayed with complete media containing 1.5%(w/v) carboxy-methylcellulose (SigmaAldrich), cultured for a further 5 days, and then fixed with 10% formalin and stained with crystal violet. Viral plaques were counted, and virus infectivity expressed as plaque-forming units per ml (PFU/ml). Infectious DENV virus titre was measured by focus forming assay as previously described [15]. Briefly, Huh7.5 cells were inoculated with dilutions of virus-containing cell culture supernatants. After 3 h cells were washed and cultured to 72 h. Cells were then fixed and labelled by indirect immunofluorescence with a ms-anti-flavivirus-IgG and anti-ms-IgG-488, to enable counting of clusters of infected cells (foci), and virus infectivity expressed as focus-forming units (FFU) per ml.

### Placental villus explant cultures

Six first trimester human placentas were used for placental villus explant cultures. Pairs of villus explants (10-20 mg) from the same placenta were co-cultured in 48 well plates on preset Matrigel^®^ (Basement membrane matrix, growth Factor reduced (gFr), ldEV-Free, Corning^®^) for 48 h to allow syncytiotrophoblast regeneration. Virus was added to duplicate wells of cultured explants, ZIKV at 10^6^PFU/ml or 10^7^PFU/ml, or DENV at 10^7^FFU/ml, and explants incubated for a further 24 h, after which virus containing media were removed, and explants gently washed twice with both PBS and DMEM media. Fresh media was added for the remaining culture period. Explants were transfected with Poly(I: C) (1 µg/ml) using lipofectamine 2000 (ThermoFisher, Invitrogen) as per the manufacturer’s instructions. Conditioned culture media and tissue samples were collected for analysis at 24, 48, 72 and 96 h post infection (hpi). At each collection, the culture media supernatant was frozen at -80 °C for plaque assay and/or ELISA analysis, while placental explant tissues were excised from Matrigel and either; embedded in OCT and frozen by immersion in an isopentane bath over liquid nitrogen, or placed into 200 µl RNAlater^®^ (ThermoFisher, Invitrogen) for 48 h before removing RNAlater^®^ supernatant and freezing for subsequent RNA isolation. Collected samples were stored at -80 °C until processing.

### Isolation of placental cytotrophoblasts

Human CTBs were isolated from first trimester placentas as previously described [16, 17]. Briefly, villous tissue was incubated 10 min at 37 °C in 10 ml PBS with 0.25%Trypsin and 200 µg/ml DNAse per gram of tissue then following PBS washing and filtering (100 μm), tissues were further digested at 4 °C overnight. Undigested tissue remaining underwent multiple PBS washes and then all supernatants were pooled into 50 ml tubes with 10%FCS and centrifuged (450xg). Cell pellets were combined into 15 ml per gram of tissue in DMEM high glucose media containing 10%FCS and 1%Antibiotic-Antimycotic solution (Sigma-Aldrich). Co-isolated fibroblasts were removed by adherence to 10 cm plastic Petri dish cells (1 h at 37 °C, 5%CO_2_/21% O_2_) and the isolated CTBs were used in infection experiments or transfected with 1 µg/ml poly(I: C) using lipofectamine 2000 (ThermoFisher, Invitrogen) as per the manufacturer’s instructions.

### RNA isolation, cDNA generation and Real-time PCR assays

Frozen RNAlater^®^ preserved explant tissues were ground with a pestle over dry ice, after which 100 µl of TriSure^®^ (Bioline) was added and further homogenisation carried out. When uniform, a further 400 µl TriSure^®^ was added and mixed well. Primary cytotrophoblasts in plates were washed with PBS and then 400 µl TriSure^®^ added to each well. Homogenised tissues and cells in TriSure^®^ were processed as per the manufacturer’s instructions for RNA isolation. RNA pellets were resuspended in 20 µl RNAse/DNAse free water (Invitrogen). First-strand synthesis of cDNA was performed as per manufacturer instructions on 8 µl of total RNA, using MMLV RNase H Minus, Point Mutant with the Recombinant RNasin^®^ Ribonuclease Inhibitor added (Promega). ISG expression was assessed as described previously [8] using the primers listed in Table 1. Results are expressed as fold change over uninfected controls from the same time point and normalised to the housekeeper gene *RPLP0*. ZIKV or DENV genome copies per µg of total RNA, were calculated using plasmid derived standard curves as described previously [8]. Plasmids were linearised (pFK-DV [12]; SacI(NEB)) (pZIKV-ICD, strain Paraiba_01/2015 [18]; EcoRV(NEB)), gel purified, and serially diluted to generate a copy number standard curve that was used to estimate viral genome copies in total RNA isolated from infected cultured villus explants and primary cytotrophoblasts.

**Table 1:** Nucleotide sequences of the primer pairs used in the quantitative real-time PCR assays performed in this study

Target gene	Forward primer	Reverse primer
*IFNβ* Interferon beta	gcagtctgcacctgaaaagatatt	tgtactccttggccttgaggta
*IFNλ1 (IL29)* Interferon Lambda 1	ggaagagtcactcaagctgaaaaac	agaagcctcaggtcccaattc
*IFNλ2/3 (IL-28 A/B)* Interferon Lambda 2/3	acatagcccagttcaagtc	gactcttctaaggcatctttg
*RPLP0* Ribosomal Protein Lateral Stalk Subunit P0	agatgcagcagatccgcat	ggatggccttgcgca
*IFI6* Interferon Alpha Inducible Protein 6	ctgaagattgcttctcttctc	cactttttcttacctgcctc
*IFIT1* Interferon Induced Protein With Tetratricopeptide Repeats 1	aacttaatgcaggaagaacatgacaa	ctgccagtctgcccatgtg
*IFIT2* Interferon Induced Protein With Tetratricopeptide Repeats 2	agaaagctgatgaggccaatg	gcatggaggctggcaaga
*IFITM1* Interferon Induced Transmembrane Protein 1	cgccaagtgcctgaacatct	cccgtttttcctgtattatctgta
*ISG15* interferon stimulated gene 15 ubiquitin like modifier	tggcgggcaacgaatt	gggtgatctgcgccttca
*MX1* Myxoma resistance Dynamin Like GTPase 1	cagcacctgatggcctatcac	catgaagaactggatgatcaaagg
*RSAD2 (Viperin)* Radical S-Adenosyl Methionine Domain Containing 2	gtgagcaatggaagcctgatc	gctgtcacaggagatagcgagaa
Zika Virus (PRVABC59)	gtgtgatgccaccatgagcta	tggcaggttccgtacacaac
Dengue Virus-2 (16681)	caatatgctgaaacgcgagaga	accagggccatgaacagtttta

### Immuno-Staining

The details of all the primary and secondary antibodies used for staining are listed in Table 2. Primary cytotrophoblasts and placental explant tissue sections were fixed with 50%acetone/50% methanol. OCT-embedded placental explant tissue sections (10 μm) on lysine coated glass slides were stored at -80 °C. Upon thawing, tissue sections were fixed, blocked with goat serum, and labelled by indirect immunofluorescence with rb-anti-ZIKV-E and ms-anti-hu-cytokeratin-7, followed by appropriate secondary antibodies. Fixed primary cytotrophoblasts were stained with either ms-anti-hu-cytokeratin-7 and rb-anti-ZIKV-E followed by appropriate secondary antibodies or, ms-anti-hu-cytokeratin-7 then gt-anti-msIgG-488, followed by ms-anti-dsRNA and gt-anti-ms-IgM. Stained samples were counter-stained with DAPI and mounted with Vectashield Antifade Mounting Medium (Vector Laboratories). Images were acquired using a Nikon TiE inverted fluorescent microscope (Nikon) as described previously [8].

**Table 2:** Antibodies used in indirect Immunofluorescence detection of ZIKV and CTB antigens

Antibody	Source
Mouse anti-human cytokeratin 7	Dako, OV-TL12730
Rabbit anti- ZIKV Envelope Protein	Genetex, GTX133314
Mouse anti-dsRNA (IgM)	3G1, hybridoma cell supernatant, Prof. Roy Hall (University of Queensland, Australia)
Mouse anti- Flavivirus E protein	Clone D1-4G2-4-1, ATCC, VR-1852
Goat Anti-mouse IgG 488	ThermoFisher, Invitrogen, **A-11,001**
Goat Anti-mouse IgG 555	ThermoFisher, Invitrogen, A-21,422
Goat anti rabbit IgG 488	ThermoFisher, Invitrogen, A-11,008
Goat anti rabbit IgG 555	ThermoFisher, Invitrogen, A-21,428
Goat anti-mouse IgM 594	ThermoFisher, Invitrogen. A-21,044

### ELISA assays

All ELISA assays were purchased through ThermoFisher Scientific Inc. VeriKine Human IFN beta ELISA Kit (41410) was manufactured by Pestka Biomedical Laboratories Inc, while the Human IL-29 ELISA Kit (BMS2049) and Human IL-28 A Elisa kit (# EHIL28A) were both produced by Invitrogen. The conditioned culture media that was collected from explant tissues was thawed on ice, and then processed on plates and absorbence data analysed as per the manufactures instructions for each interferon assay.

### Statistical analysis

All graphing and statistical analyses of data were performed using GraphPad Prism 8.0. mRNA qRT-PCR and viral assay data were log [10] transformed prior to statistical analysis. The statistical tests used are indicated in the Figure legends.

## Results

### ZIKV infects human first trimester placental explants

**Fig. 1:**
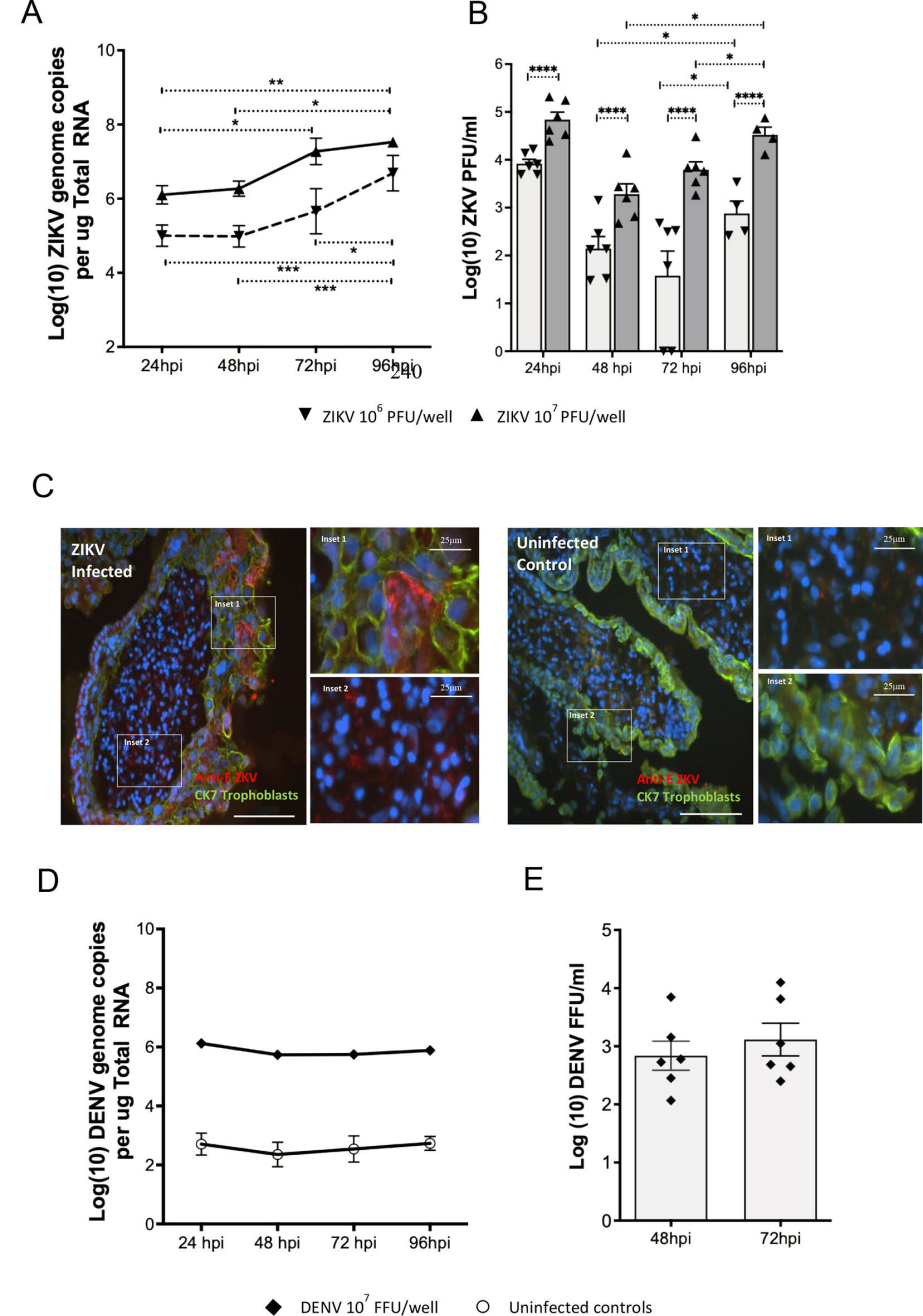
ZIKV infects and replicates in first trimester placental explants. First trimester placental explant tissue cultures (*n* = 6 patients in duplicate) were infected with ZIKV at 10^6^ or 10^7^ PFU/well and (**A**) ZIKV RNA genomes quantified by qRT-PCR. Data were log transformed and analysed by Ordinary Two Way ANOVA (* *P* < 0.05, ** *P* < 0.01. *** *P* < 0.005) or (**B**) infectious ZIKV particles in culture supernatant from infected explant tissues detected by plaque assay. Some explants that were infected at 10^6^ PFU/well did not produce infectious plaques at 72hpi. The 24hpi data represents the viral inoculum, as virus was left on explants for 24 h. This data was not included in subsequent statistical analysis. Data were log transformed and analysed by Ordinary Two-Way ANOVA. (* *P* < 0.05, **** *P* < 0.0001). (**C**) Frozen sections from ZIKV infected explant tissues (96hpi) were stained for ZIKV-Envelope antigen (red), cytokeratin (green) and nuclei (DAPI). Bars represent 100 μm unless otherwise indicated. Images are representative of multiple samples analysed (**D).** First trimester placental explant tissue cultures (6 patients in duplicate) were infected with DENV and at timepoints indicated DENV RNA genomes quantified by qRT-PCR including in uninfected controls. (**E**) infectious DENV particles in the culture supernatant from infected explant tissues detected by focus forming assay. All data are means± SE

In this study we infected first trimester placental explant tissues from six patients (in duplicate) with two *orthoflavivirus* family members ZIKV and DENV. First trimester placental explants were permissive for ZIKV infection displaying a significant increase in ZIKV RNA from 48-96hpi and significant increases in production of infectious virus (PFU/ml) from 48 to 96hpi at both inoculum levels (Fig. 1A, B). The levels of infectious virus were significantly higher at all time points in the 1 × 10^7^PFU/ml samples compared to those inoculated at 1 × 10^6^PFU/ml (Fig. 1B). Furthermore, at 72hpi only 4 of 6 explants inoculated with 1 × 10^6^PFU/ml produced infectious virus (plaque assay), while all explants inoculated at 1 × 10^7^PFU/ml produced infectious virus. Morphological changes due to viral infection were not observed in any cultured placental explants. To further validate infection status, we also demonstrate ZIKV-E-antigen in the stroma and syncytiotrophoblast layer of infected placental explants (Fig. 1C). In contrast infection of placental explant tissues with DENV did not result in productive infection (Fig. 1D, E). Collectively, these results demonstrate that first trimester placental explant cultures can support ZIKV replication.

Figure 1.

### ZIKV infects isolated human primary trophoblasts

**Fig. 2:**
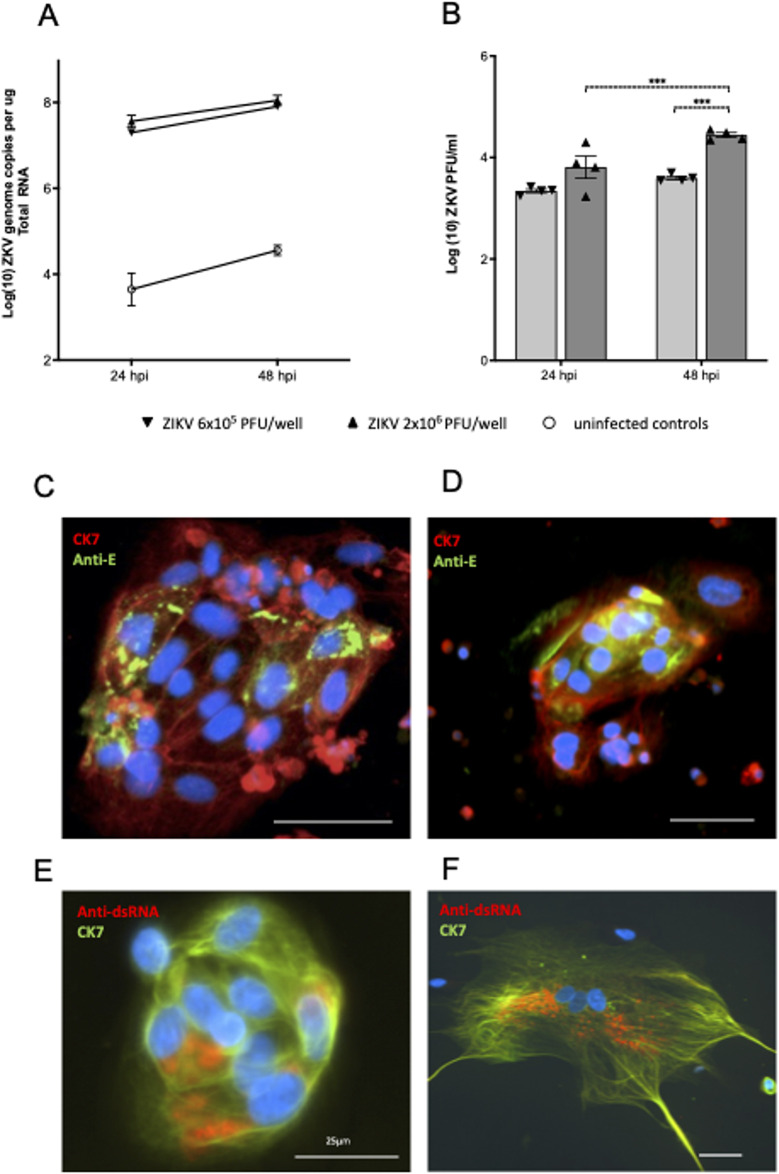
Zika virus infects and replicates in isolated first trimester placental trophoblasts. (**A**) Trophoblasts from first trimester placental samples were infected with ZIKV then at 24hpi and 48hpi ZIKV genome RNA was quantified using qRT-PCR, including in uninfected control cells. Data are means ± SE, *n* = 3 patient samples. (**B**) Infectious ZIKV in CTB culture supernatant was quantified by plaque-assay. Data are means ± SE, *n* = 2 patient samples in duplicate. Data were log transformed and analysed by Ordinary Two Way ANOVA (*** *P* < 0.005). (**C-D**) Isolated trophoblasts were fixed and stained for the CTB marker, cytokeratin-7 (red) and ZIKV-E antigen (green). (**E**,** F**) Detection of ZIKV genome dsRNA replication intermediates used anti-dsRNA Ab (red), cytokeratin 7 (green) and nuclei (DAPI). Bars represent 50 μm unless otherwise indicated

We next infected primary CTBs and STBs with ZIKV and detected, viral RNA (qRT-PCR) in infected trophoblast cells at 24hpi and 48hpi, and infectious virus in harvested culture media at both time points (Fig. 2A, B). No significant increase in ZIKV RNA was observed over time, most likely due to the small number of isolated CTBs present. Nevertheless, the corresponding plaque assay revealed significantly more infectious virus recovered at 48hpi compared to 24hpi (Fig. 2B), indicative of active viral replication.

To confirm infectious viral production, primary CTBs were also stained for ZIKV-E-antigen (ZIKV-E-Ag), dsRNA and cytokeratin-7 (trophoblast marker). Most isolated cells were cytokeratin-7 positive with a proportion also displaying ZIKV-E-Ag or dsRNA positive staining localised to the cytoplasm, as expected for ZIKV infection (Fig. 2C, D, E). CTBs were also cultured for > 4 days resulting in the spontaneous generation of STBs, evident by the formation of large multinucleated cells. STBs were infected with ZIKV at MOI-5.0 and stained for cytokeratin-7 and dsRNA (Fig. 2F) suggesting active viral replication in multinucleated STBs.

Together, our quantitative PCR for ZIKV genomes and visualisation of ZIKV-E-Ag and dsRNA positive punctate structures reminiscent of ZIKV replication vesicles, confirm that ZIKV can infect both primary derived CTB and their syncytialised derivatives.

### ZIKV infection induces IFN and ISG expression in human first trimester placental explants

**Fig. 3:**
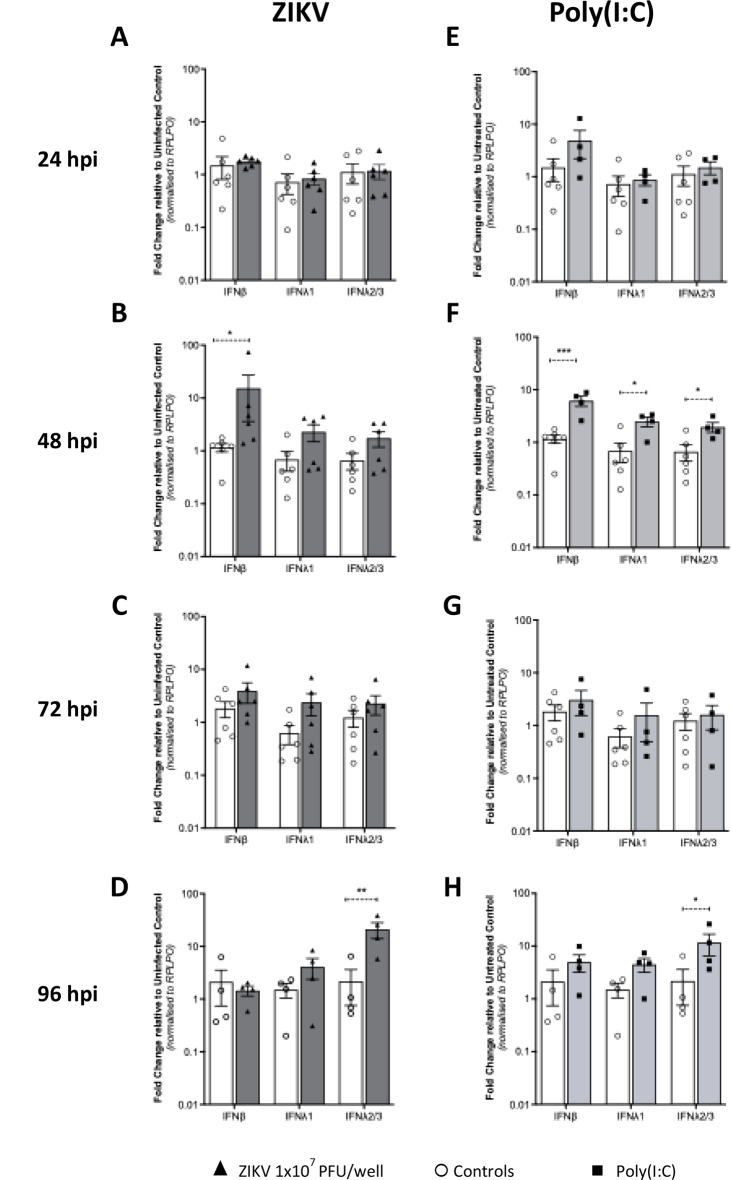
ZIKV infection induces a Type III IFN response in first trimester placental explant tissue. Explant tissue was (**A**,** B**,**C**,** D**) infected with 10^7^/ml ZIKV or (**E**,** F**,**G**,** H**) stimulated with 1 µg/ml poly(I: C)(**A**,** B**,**C** are *n* = 6 patients) (**D**,** E**,**F**,** G**,**H** are *n* = 4 patients). RNA was collected 24, 48, 72 and 96hpi followed by qRT-PCR analysis for the detection of *IFNβ*, *IFNλ1* and *IFNλ2/3*. Data are normalised to the housekeeping gene *RPLP0* and expressed as a fold-change relative to mock-infected control at each timepoint. (data are means ± SE) * *P* < 0.05, ** *P* < 0.01 ***< P0.005. Statistical analysis was performed on log transformed data at each time point using multiple unpaired T test for each gene

**Fig. 4:**
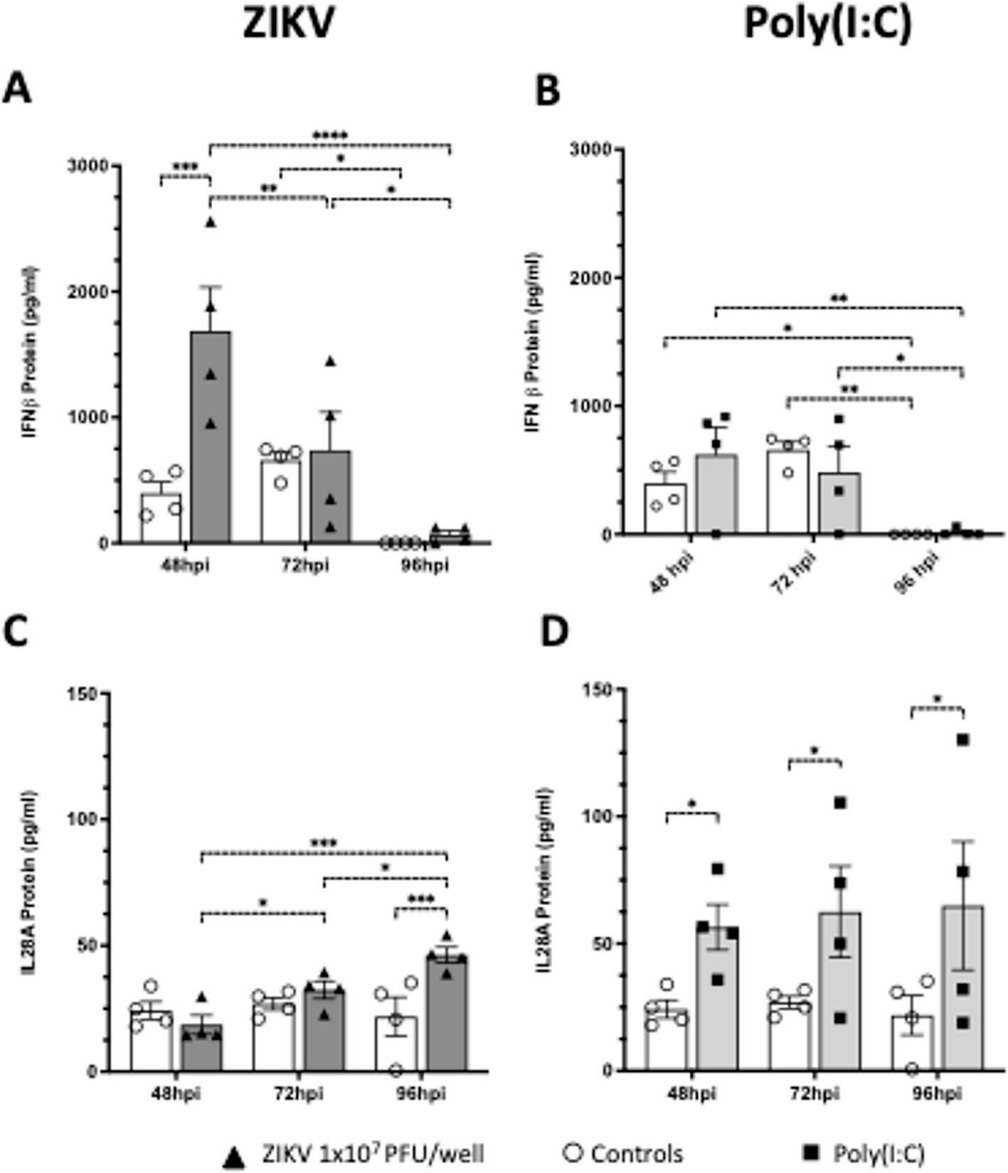
ZIKV infection induces an IFN protein in response in first trimester placental explant tissue. Explant tissue was infected with 10^7^/ml ZIKV (**A, C**) or stimulated with 1 µg/ml poly(I: C) (**B**,** D**) (*n* = 4 patients, data are means ± SE. *P < 0.05, **P < 0.01, ***P < 0.005, ****P < 0.0001). Conditioned culture media was collected from cultured explants at 48, 72 and 96hpi and then analysed by ELISA assay for hIFNβ and hIL-28 A (I FNλ2). Statistical analysis was performed using a 2-way ANOVA

We next investigated Type I and III IFN mRNA expression following ZIKV infection of first trimester placental explants. At 24hpi there was no increase in IFN mRNAs, suggesting a period of establishment of viral replication. However, at 48hpi there was a significant increase in IFNb mRNA that declined thereafter (Fig. 3A-D). Interestingly, the expression of IFNλ2/3 increased at 96hpi suggesting a biphasic pattern of IFN expression with IFNβ expressed early and IFNλ2/3 expressed late in infection. To verify these responses, we next measured Type I and III IFN protein levels in the conditioned media. IFNβ protein levels mirrored the changes seen in mRNA (Fig. 4A) including the decline from 72hpi to 96hpi, where it became almost undetectable (Fig. 4A). Interleukin-29 (IFNλ1) levels were below the level of detection of the ELISA assay (15pg/ml). Interleukin 28 A (IFNλ2) protein levels in ZIKV infected explants also correlated with the changes mRNA (Fig. 4C).

To assess placental explant innate immune competency, we also measured IFN mRNA expression following treatment of explant cultures from 4 placentas (in duplicate) with Polyinosinic–polycytidylic acid (poly(I: C)), a synthetic double stranded RNA (viral RNA mimic) that is well documented to activate cytosolic pattern recognition receptors and induce IFN expression both in vitro and in vivo. Poly(I: C) induced expression of all IFNs at 48 h reflecting direct stimulation of PPRs that is independent of viral replication, while at 96 h poly(I: C), like viral infection, IFNλ2/3 mRNA expression was upregulated (Fig. 3E-H). Interestingly, IFNβ protein levels measured at 48hpi were higher than those seen following 1 µg/ml poly(I: C) stimulation (Fig. 4B) while levels of IL-28 A(IFNλ2) were elevated over control by poly(I: C) treatment at all time points but did not fluctuate, suggesting saturation of receptor activation (Fig. 4D). These results confirm the ability of the first trimester placenta to respond at the innate immune level to ZIKV infection and suggest that Type I and Type III IFN responses are regulated in a biphasic nature.

**Fig. 5:**
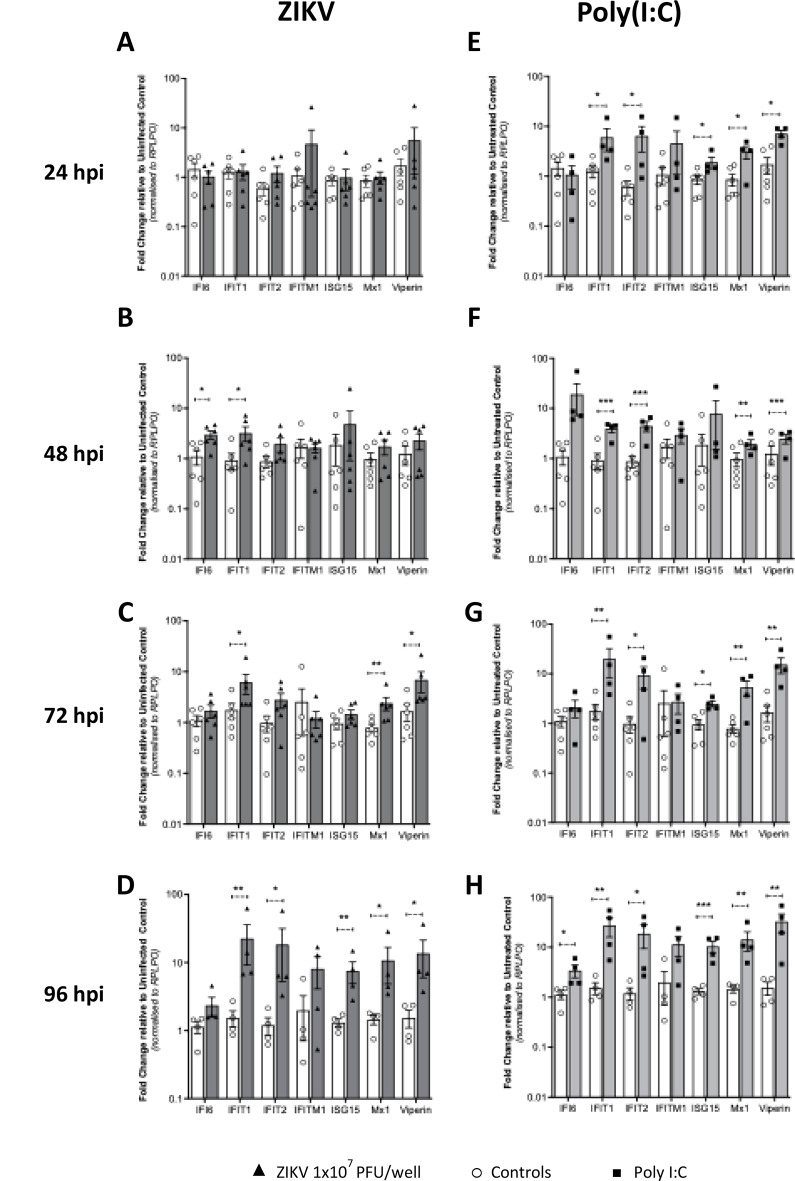
ISG responses in first trimester placental explant tissue. Explant tissue was (**A**,** B**,**C**,** D**) infected with 10^7^/ml ZIKV or (**E**,** F**,**G**,** H**) stimulated with 1 µg/ml poly(I: C)(**A**,** B**,**C** are *n* = 6 patients, **D**,** E**,**F**,** G**,**H** are *n* = 4 patients. RNA was collected 24, 48, 72 and 96hpi followed by qRT-PCR analysis for the detection of antiviral ISGs. Data are normalised to the housekeeping gene *RPLP0* and expressed as a fold-change relative to mock-infected control at each timepoint (data are means ± SE) * *P* < 0.05, ** *P* < 0.01 ***< P0.005. Statistical analysis was performed on log transformed data at each time point using multiple unpaired T test for each gene

As Type I and Type III IFNs mediate their antiviral effects through expression of ISGs, we next evaluated mRNA expression of several well-characterised antiviral ISGs (*IFI6*,* IFIT1*,* IFIT2*,* IFITM1*,* ISG15*,* Mx1 and Viperin*) known to be induced by either Type I or III IFNs. Like IFNβ mRNA expression, we observed no increase in ISG expression at 24hpi, however at 48hpi there was a modest, but significant, increases for IFI6 and IFIT1 only. The peak of ISG mRNA expression occurred at 96hpi and correlated with Type III IFN expression (Fig. 5A-D). A similar observation was noted with stimulation of explants with poly(I: C) in which ISG mRNA expression correlated with Type I and III IFN expression with maximal expression across all ISG seen at 96hpi (Fig. 5E-H). Collectively, these results suggest that first trimester placental explants can respond to poly(I: C) and ZIKV infection at the IFN and ISG mRNA expression level, and that the antiviral response following infection is driven by the Type III IFNs late in infection.

### ZIKV infection induces IFN and ISG mRNA expression in isolated human primary trophoblasts

**Fig. 6:**
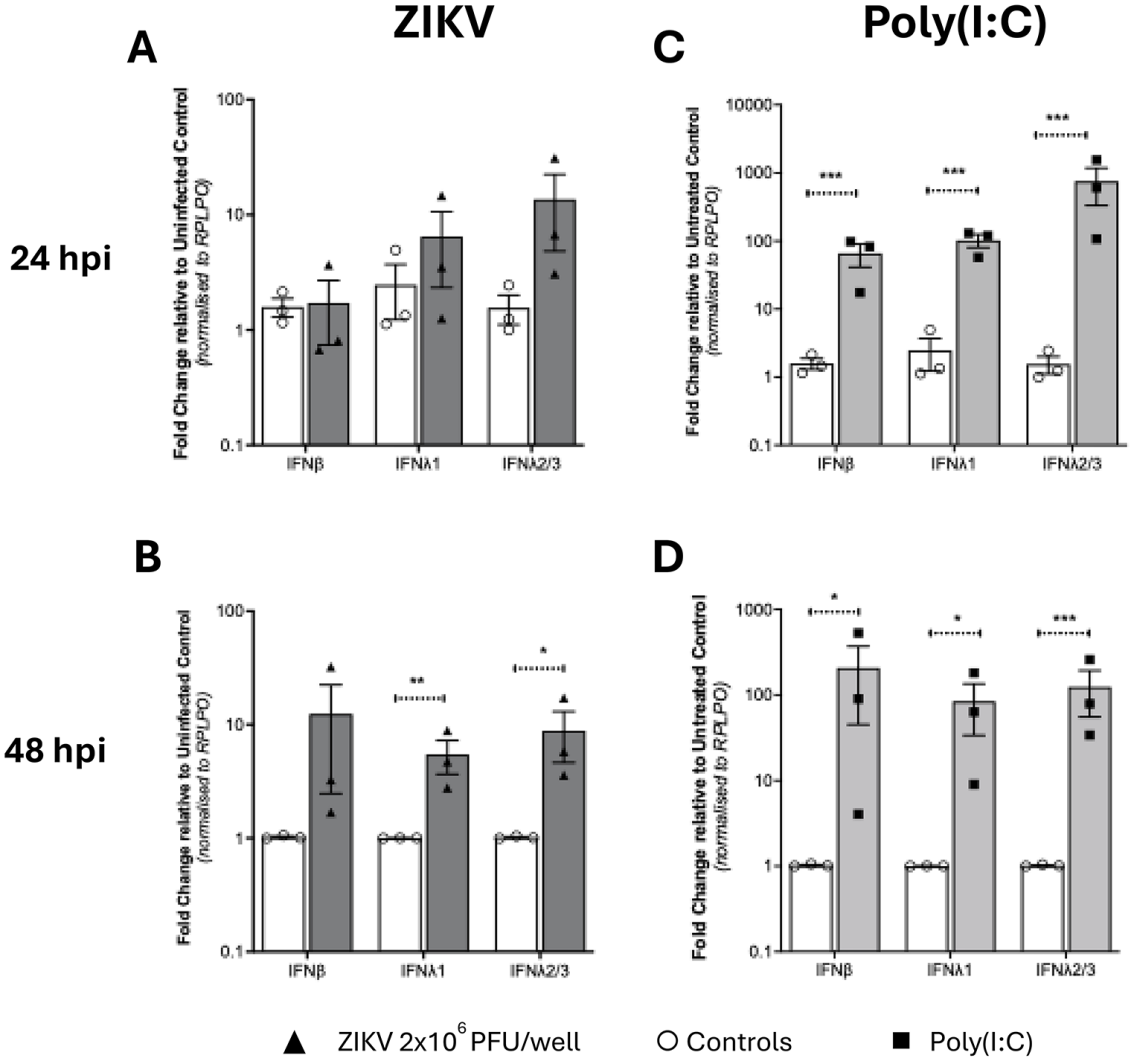
Interferon mRNA expression in isolated first trimester placental trophoblasts. Trophoblasts isolated from first trimester placental samples were (**A and B**) infected with ZIKV MOI-5 or (**C and D**) stimulated with 1 µg/ml poly(I: C) and RNA collected at 24 h and 48 h (*n* = 3 patient samples in duplicate). qRT-PCR was then used to detect *IFNβ*,* IFNλ1* and *IFNλ2/3* mRNA. Data are normalised to the housekeeping gene *RPLP0* and expressed as a fold-change relative to the mock-infected control at each timepoint (data are means ± SE). Statistical analysis was performed on log transformed data at each time point using multiple unpaired T test for each gene

**Fig. 7:**
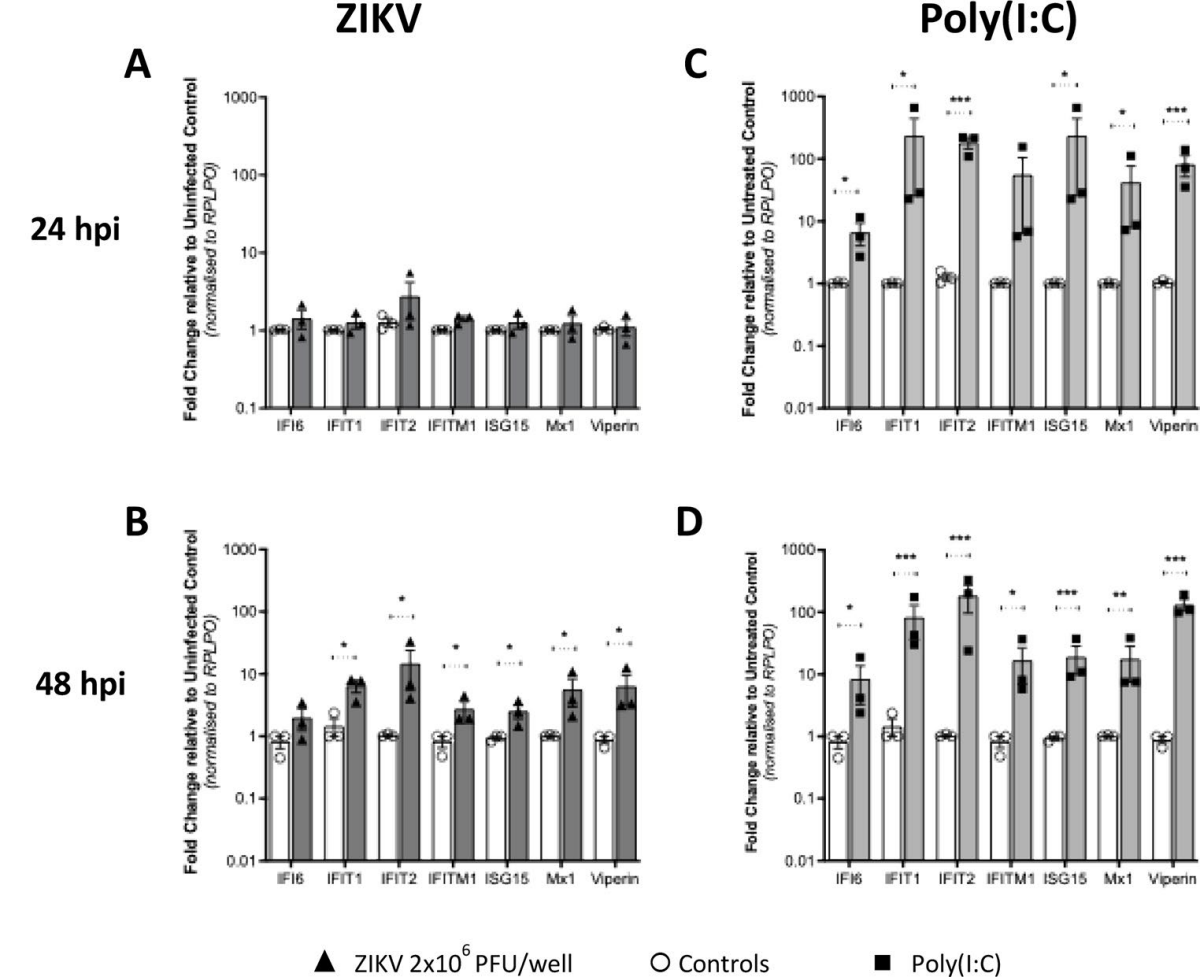
ISGs mRNA expression in isolated first trimester placental trophoblasts. Trophoblasts isolated from first trimester placental samples were (**A and B**) infected with MOI-5 ZIKV or (**C and D**) stimulated with 1 µg/ml poly(I: C) and RNA collected at 24 h and 48 h (*n* = 3 patients). qRT-PCR was then used to detect mRNA for antiviral ISGs. Data are normalised to the housekeeping gene *RPLP0* and expressed as a fold-change relative to the mock-infected control at each timepoint (data are means ± SE). Statistical analysis was performed on log transformed data at each time point using multiple unpaired T test for each gene

Cytotrophoblasts isolated from first trimester tissue (3 patients in duplicate) were infected with ZIKV (MOI-1.0 and − 5.0) and processed for quantitation of mRNA for antiviral ISGs using qRT-PCR. At 24hpi of CTBs there was no significant increase in expression of IFNλ2/3 (Fig. 6A), or ISG mRNAs (Fig. 7B). However, at 48hpi when CTBs were productively infected, we noted significant increase in mRNA expression for IFNs (Fig. 6B). Following poly(I: C) treatment, CTBs responded with a robust induction of IFNβ, IFNλ1 and λ2/3 at 24hpi that was maintained at 48hpi post stimulation (Fig. 6C, D).

Consistent with our IFN mRNA expression data following ZIKV infection, we observed an increase for all antiviral ISG mRNAs, at 48hpi but not 24hpi (Fig. 7A, B). In contrast, all ISGs were significantly expressed at 24 h and 48 h post poly(I: C) stimulation at both time points, reflecting the potent nature of poly(I: C) to induce innate responses (Fig. 7A, B). These results suggest that human primary CTBs are permissive to ZIKV infection and have the appropriate pattern recognition receptors and signalling adaptor molecules to sense viral RNA and activate the downstream induction of mRNAs for type I and III IFN and antiviral ISGs.

## Discussion

ZIKV infection in early pregnancy can have a severe impact on fetal development [4, 19–21]. As such there is a need to understand how this *orthoflavivirus* can cross the placental barrier to infect the developing fetus. Both in vivo and in vitro models have been developed in attempts to understand ZIKV replication and placental responses [22–25]. However, due to the complex nature of the human placenta, many of these studies do not truly represent physiological infection in humans. Studies investigating ZIKV infection and host response of tissues and cells derived from human first trimester placentas are essential if we are to understand the complex relationship between virus and host. Despite this, there are only limited numbers of studies in this niche, which is indicative of the difficulties in obtaining and culturing first trimester placental samples. In this report, we show that the Puerto Rico strain of ZIKV (PRVABC-59) readily replicates within villus explants and isolated trophoblasts from human first trimester placentas, while the related *orthoflavivirus* DENV does not. Furthermore, we demonstrate that these tissues and cells can respond to viral infection with the production of IFNs and ISGs but do so in a temporal manner.

We investigated ZIKV infection in first trimester placental explants from 6 donors over 96 h of culture and demonstrated active replication in all donor samples by increases in, ZIKV RNA (qRT-PCR), production and release of infectious virus (into the culture supernatant), and detection of ZIKV-E antigen in trophoblasts and stromal cells. These results suggest active spread of ZIKV throughout the placental explants and a transmission pathway across the endothelium into fetal blood. Our observations are in concordance with previous studies, even though they have used fewer first trimester placenta donor samples [10, 26–29]. ZIKV infection of trophoblasts from first trimester placentas [30], human stem cell derived trophoblasts and trophoblast organoids [31], have been reported and we were also able to productively infect purified human first trimester trophoblasts. We did however extend this to infection of primary culture-induced STBs, an important finding due to the critical barrier role of the STB in pathogen transfer from the mother to the fetus. Combined our evidence suggests that CTBs and STBs derived from first trimester tissues are both permissive for ZIKV infection with implications for placental function, development, and transmission of infection from the mother to the fetus. This is important as the villus STB layer directly contacts maternal ZIKV infected blood. Additionally, there is previously published evidence of differences in infection susceptible across gestation. Undifferentiated trophoblast stem cells are more susceptible to infection than differentiated STB or EVTs, implying increased susceptibility of cells of the early placenta to ZIKV infection [31]. Whilst ZIKV infection of mid-gestation and term placental explants has been demonstrated [32–36], infection early in gestation is reportedly more prolific than that seen later in gestation [10]. The reason for this in unclear but it has been suggested that embryonic stem cell derived trophoblasts express a limited basal repertoire of antiviral ISGs when compared to isolated term trophoblasts [11], while term trophoblasts have been shown to resist ZIKV infection through the production of the type III interferon, IFNλ1 [37].

The host innate response to viral infection controls viral infection through the expression of predominantly type I IFNs and antiviral ISGs. This potent, rapid and transient type I inflammatory response is actually detrimental to pregnancy in mouse models [34], and results in formation of syncytial knots and actin filament disassembly in human placental explants an effect not observed in IFNλ treated explants [38, 39]. Type III IFNs (IFNλ) are known to control infection at mucosal surfaces and there is mounting evidence these IFNs are more important in a placental response to infection than type I IFNs [40–43]. In first trimester placental tissues infected with ZIKV, we observed an IFNβ peak at 48hpi, followed by a switch to expression of IFNλ2/3 later in infection at 96hpi, concomitant with expression of antiviral ISG mRNAs. Infection of primary CTBs resulted in increased mRNA expression of IFNλs. Moreover, we showed similar results in explant tissue and isolated CTBs that were stimulated with the dsRNA analogue poly(I: C). Collectively these results indicate that the early gestation human placenta can respond to viral infection in a biphasic manner, initially with a type I IFN response followed by type III IFN expression. Type III interferon has been previously shown to by us and other to inhibit and control ZIKV replication [13, 44].

Type I and III IFNs initiate STAT1/2 phosphorylation and ISG responses through different receptor complexes with distinct expression profiles [45, 46]. The type I receptor (IFNαR1/IFNαR2) is expressed ubiquitously while the type III receptor (IFNλR1/IL-10Rβ) is limited to mucosal and epithelial cells such as those found in the placenta. Previous studies have revealed a unique IFNλ gene profile compared to that of IFNβ in primary female reproductive tract (FRT) cell lines [47]. FRT cell lines show that while type I and III IFNs induce similar ISG profiles, the type III IFNλ response is less proinflammatory and more antiviral than the type I IFN response [13]. Thus, it is likely the expression of IFNλ in the early placenta, rather than IFNα/β, provides a balance between limiting infection, restricting inflammation and preserving pregnancy.

In both ZIKV infected placental explants and isolated CTBs, we saw significant upregulation of ISGs with antiviral function [48, 49]. However, the ISG response in CTBs does not reach the levels of that seen in the explants, likely due to the limited early time points we have studied. Most interestingly, we saw upregulation of *IFI6*, an established anti-*orthoflavivirus* effector protein that restricts the formation of endoplasmic reticulum membrane associated viral replication complexes [50], and *Viperin* which targets the ZIKV NS3 protein for degradation [51]. Previous reports have suggested that the response of the first trimester placenta to ZIKV infection is a type I IFN response [30], but here we show a delayed Type III IFN response and concomitant antiviral ISG expression. A similar delay in ISG expression has been previously observed in ZIKV infected primary trophoblasts [30, 41], and in placental cell lines [52–54]. However, in these studies expression of type III IFNs was not reported. Although these differences may be related to cell type, antagonism of the interferon response by ZKV has also been previously reported others. ZIKV proteins can antagonise IFN signalling mediators Stat1 and Stat2 to impact on downstream ISG expression [9, 55]. This ISG expression pattern necessitates careful interpretation of studies using cell lines or explant tissues at restricted time points (i.e., 48hpi only) as they may not reflect authentic responses of the placenta to infection. Our studies using ZIKV infection of first trimester placental explant tissue accurately reflect the complex cellular composition of the placenta and suggest that a delayed type III IFN response and ISG expression to infection is physiological. This has implications for infection outcome as a delayed and less potent type III antiviral response, while important to maintain pregnancy, would provide a window to establish ZIKV infection and transmission to the developing fetus.

In summary, our work supports previous published research suggesting that during early pregnancy there is a fine balance between a viral induced antiviral response and a detrimental impact on the developing placenta and fetus. We have shown that ZIKV can productively infect first trimester placental explants and primary trophoblasts inducing a delayed type III IFN response (IFNλ2/3) to ZIKV infection that corresponds to expression of antiviral ISGs. This delayed response is likely to have a lower propensity to induce inflammation and antiviral ISGs in comparison to a type I IFN responses and may explain the inability of the first trimester placenta to limit viral infection. Further work is required to characterise the innate responses and susceptibly of the placenta to infection across all stages of gestation to understand the balance between the antiviral response and inflammation, that determines dissemination of virus to the fetus and infection outcome.

## Data Availability

The quantitative real-time PCR gene expression and ELISA Assay datasets used and/or analysed during the current study are available from the corresponding author on reasonable request.
