# Correction: The first trimester human placenta responds to Zika virus infection inducing an interferon (IFN) and antiviral interferon stimulated gene (ISG) response

**DOI:** 10.1186/s12985-026-03157-7

**Published:** 2026-04-22

**Authors:** Kylie H. Van der Hoek, Tanja Jankovic-Karasoulos, Dylan McCullough, Rosa C. Coldbeck-Shackley, Nicholas S. Eyre, Claire T. Roberts, Michael R. Beard

**Affiliations:** 1 Research Centre for Infectious Diseases, The University of Adelaide, Adelaide, SA 5005 Australia; 2 School of Biological Sciences, The University of Adelaide, Adelaide, SA 5005 Australia; 3 University of Adelaide, The Robinson Research Institute, Adelaide, SA 5005 Australia; 4 College of Medicine and Public Health, Flinders University, Flinders Health and Medical Research Institute, Adelaide, SA 5005 Australia; 5 Department of Molecular and Biomedical Science and Research Centre for Infectious Diseases, University of Adelaide, Adelaide, SA 5005 Australia

**Correction: Virology Journal (2025) 22:108**

10.1186/s12985-025-02729-3

In this article [1], Fig. 6 appeared incorrectly and have now been corrected in the original publication. For completeness and transparency, both correct and incorrect versions are displayed below.

The original article has been corrected.

Incorrect Fig. 6.

**Fig. 6:**
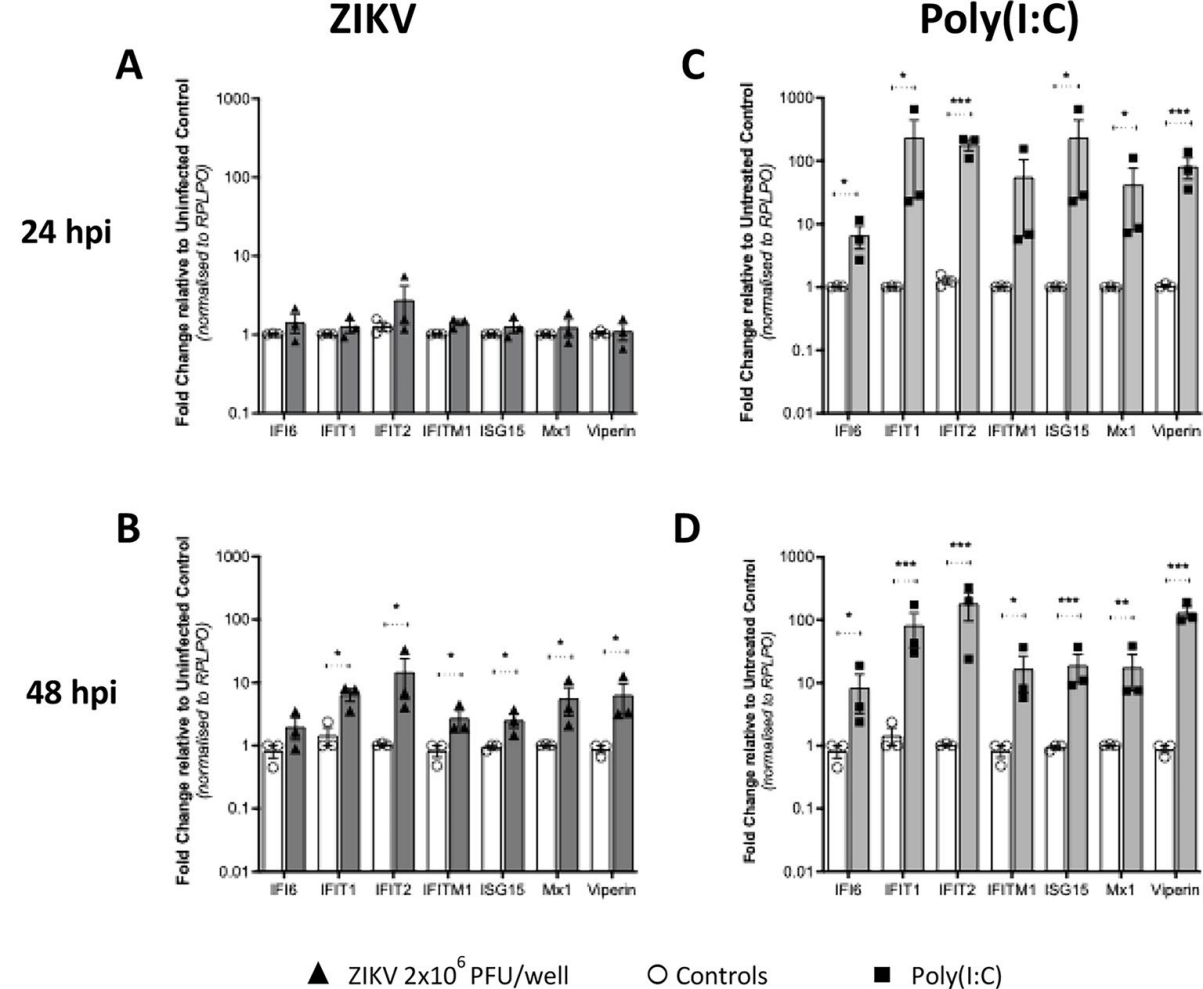
Interferon mRNA expression in isolated first trimester placental trophoblasts. Trophoblasts isolated from first trimester placental samples were (**A** and **B**) infected with ZIKV MOI-5 or (**C** and **D**) stimulated with 1 µg/ml poly(I: C) and RNA collected at 24 h and 48 h (n = 3 patient samples in duplicate). qRT-PCR was then used to detect IFNβ, IFNλ1 and IFNλ2/3 mRNA. Data are normalised to the housekeeping gene RPLP0 and expressed as a fold-change relative to the mock-infected control at each timepoint (data are means ± SE). Statistical analysis was performed on log transformed data at each time point using multiple unpaired T test for each gene

Correct Fig. 6.

**Fig. 6:**
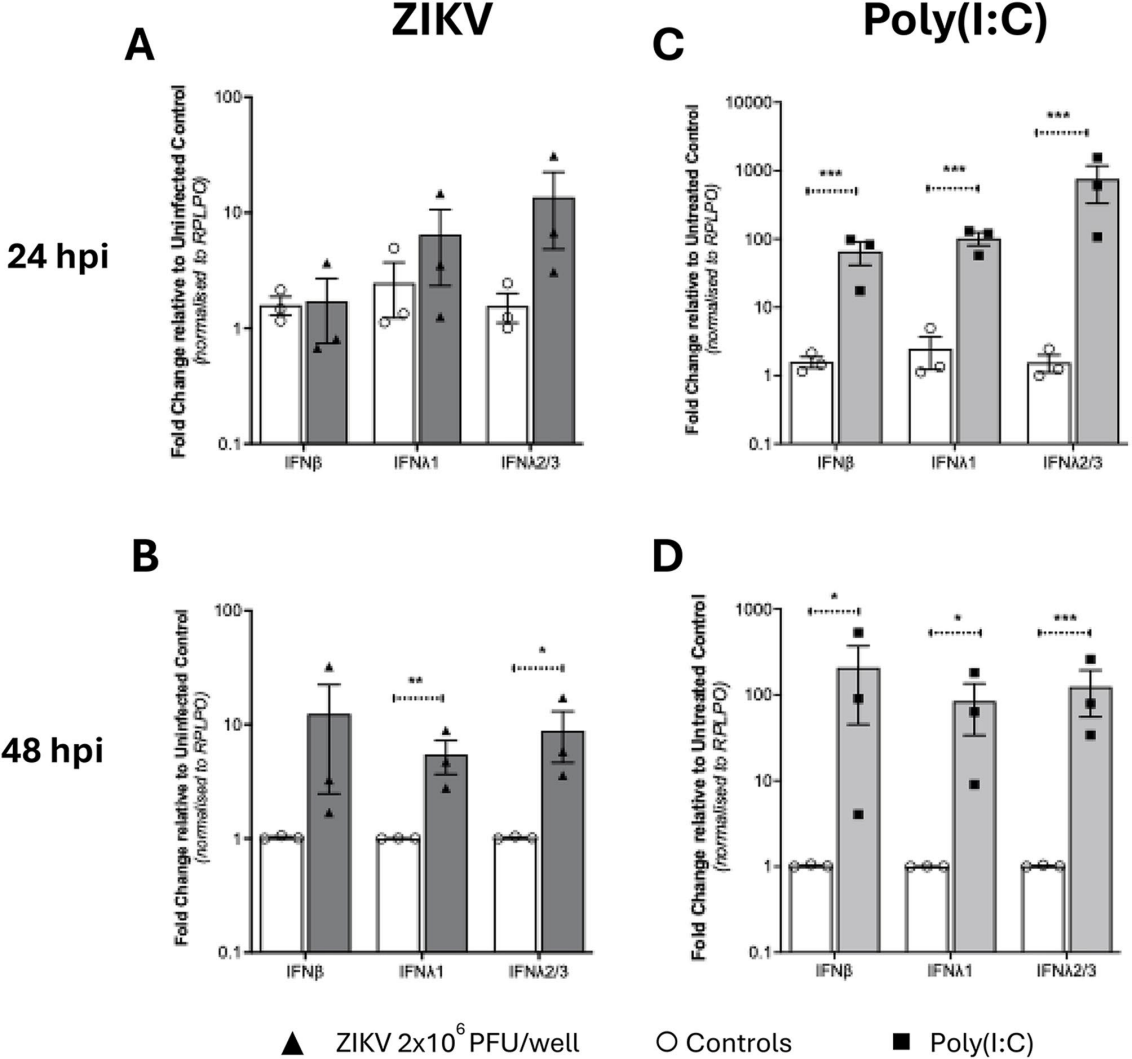
Interferon mRNA expression in isolated first trimester placental trophoblasts. Trophoblasts isolated from first trimester placental samples were (**A** and **B**) infected with ZIKV MOI-5 or (**C** and **D**) stimulated with 1 µg/ml poly(I: C) and RNA collected at 24 h and 48 h (*n* = 3 patient samples in duplicate). qRT-PCR was then used to detect IFNβ, IFNλ1 and IFNλ2/3 mRNA. Data are normalised to the housekeeping gene RPLP0 and expressed as a fold-change relative to the mock-infected control at each timepoint (data are means ± SE). Statistical analysis was performed on log transformed data at each time point using multiple unpaired T test for each gene
